# Availability and affordability of medicines and diagnostic tests recommended for management of asthma and chronic obstructive pulmonary disease in sub-Saharan Africa: a systematic review

**DOI:** 10.1186/s13223-019-0329-2

**Published:** 2019-03-07

**Authors:** Davis Kibirige, Richard E. Sanya, Rebecca Nantanda, William Worodria, Bruce Kirenga

**Affiliations:** 1 Department of Medicine, Uganda Martyrs Hospital Lubaga, P.O.BOX 14130, Kampala, Uganda; 2 Non-communicable Diseases Theme, Medical Research Council/Uganda Virus Research Institute and London School of Hygiene and Tropical Medicine Uganda Research Unit, Entebbe, Uganda; 3 Makerere University Lung Institute, Makerere University College of Health Sciences, Kampala, Uganda; 4 Department of Paediatrics and Child Health, Makerere University College of Health Sciences, Kampala, Uganda; 5 Division of Pulmonology, Department of Medicine, Mulago National Referral and Teaching Hospital, Kampala, Uganda

**Keywords:** Availability, Affordability, Essential medicines, Diagnostic tests, Asthma, Chronic obstructive pulmonary disorders, COPD, Sub-Saharan Africa, Africa

## Abstract

**Background:**

Early accurate diagnosis and sustainable availability of affordable medicines and diagnostic tests is fundamental in optimal management of asthma and chronic obstructive pulmonary disease (COPD). We systematically reviewed original research articles about availability and affordability of medicines and diagnostic tests recommended for management of asthma and COPD in sub-Saharan Africa (SSA).

**Methods:**

We searched PubMed, Scopus and African Journal Online for original research articles conducted in SSA between 2000 and March 2018 containing information about availability and affordability of any recommended medicine and diagnostic test for asthma and COPD.

**Results:**

The search yielded 9 eligible research articles. Availability of short-acting beta agonists (SABA), inhaled corticosteroids (ICS) and short acting anti-muscarinic agents (SAMA) ranged between 19.9–100%, 0–45.5% and 0–14.3% respectively. Combination of ICS-long acting beta agonists (LABA) were available in 0–14.3% of facilities surveyed. There was absence of inhaled long acting anti-muscarinic agents (LAMA) and LAMA/LABA combinations. Spirometry and peak expiratory flow devices were available in 24.4–29.4% and 6.7–53.6% respectively. Affordability of SABA and ICS varied greatly, ranging from < 2 to 107 days’ wages while ICS–LABA combinations, SAMA and oral theophylline plus leukotriene receptor antagonists cost 6.4–17.1, 13.7 and 6.9 days’ wages respectively.

**Conclusion:**

Availability and affordability of medicines and diagnostics recommended for the management of asthma and COPD is a big challenge in SSA. Research about this subject in this region is still limited. More robustly performed studies are required to further understand the magnitude of inequity in access to these medicines and diagnostic tests in SSA and also to formulate simple pragmatic solutions to address this challenge.

## Background

### Burden of asthma and COPD: Globally and in sub-Saharan Africa

The global burden of chronic respiratory disorders (CRD) particularly asthma and chronic obstructive pulmonary disease (COPD) continues to increase especially in low-and middle income countries (LMIC), posing a substantial public health threat [1]. The 2015 Global Burden of Diseases, Injuries and Risk Factors (GBD) study reported an increase in the prevalence of asthma and COPD by 12.6% and 44.2% respectively from 1990 to 2015. This was associated with an increased rate of mortality due to COPD [2]. In 2015, 3.2 million people and 0.4 million people died from COPD and asthma worldwide respectively. This represents an 11.6% increase in COPD-related deaths and a 26.7% decrease in asthma-related deaths when compared to estimates in 1990 [2]. The majority of these deaths were reported in LMIC. Smoking (both active and passive), ambient particulate matter, household pollution and occupational triggers were identified as the key risk factors and contributors to DALYS for both asthma and COPD [2].

Increasing trends of morbidity due to asthma and COPD have also been reported in sub-Saharan Africa (SSA). Regarding the prevalence of asthma in Africa, a systematic review by Adeloye et al. [3] of 45 relevant studies published between 1990 and 2012 estimated about 74.4 million, 94.8 million and 119.3 million asthma cases in the total population in 1990, 2000 and 2010 respectively. A higher crude prevalence of asthma was noted in the urban areas compared to the rural areas.

A systematic review conducted in 2011 by Finney et al. [4] reported estimates of COPD to vary between 4 and 25%. This data was obtained from 9 studies that were performed in 4 countries (Nigeria, South Africa, Malawi and Cape Verde). Only one study used a population based representative sampling approach. Another systematic review performed by Adeloye et al. [5] in 2012 that included 13 eligible studies (5 of which were based on spirometry data) reported a prevalence of COPD of 13.4% using spirometric data and 4% using non spirometric data. The most recent community surveys that have been conducted between 2013 to-date to ascertain the burden of COPD in Cameroon, Malawi, Nigeria, Uganda and Tanzania have reported prevalence of 2.4%, 7.7%, 4.2%, 16.2% and 17.5% respectively [6–10].

To effectively address the public health concern that asthma and COPD pose in SSA, health systems should be well structured to prevent, diagnose early and optimally manage these conditions. Consistent availability of affordable medicines and diagnostic tests is a fundamental component in the management of asthma and COPD in clinical practice. The Global Initiative for Asthma (GINA) and Global Initiative for Chronic Obstructive Lung Disease (GOLD) guidelines recommend the use of inhaled short acting beta agonists (SABA), inhaled short acting anti muscarinic agents (SAMA), inhaled long acting anti muscarinic agents (LAMA), inhaled long acting beta agonists (LABA) and LAMA combinations, inhaled corticosteroid (ICS)–LABA combinations, ICS monotherapies, oral methylxanthines, oral leukotriene receptor antagonists (LTRA), pneumococcal vaccination and phosphodiesterase-4 inhibitors as the mainstay pharmacological therapies in the management of asthma and COPD [11, 12].

Citing the evident increase in morbidity and mortality due to asthma and COPD in SSA, it is essential to document the current status of availability and affordability of these medicines and diagnostic tests recommended for management of asthma and COPD in SSA. This will help in the guiding the formulation and implementation of pragmatic solutions to address the challenges of poor availability and high cost of these medicines and diagnostic tests.

Availability and affordability of medicines and diagnostic tests recommended for the management of asthma and COPD in SSA has not been systematically studied to-date. We, therefore undertook a systematic review of relevant original studies performed between 2000 and March 2018 that investigated availability and affordability of these medicines and diagnostic tests in SSA.

## Methods

A comprehensive literature search of PubMed, Scopus and African Journal Online was performed for original research articles in English language performed between 2000 and March 2018 with an objective of determining the scope of availability and affordability of key medicines and diagnostic tests recommended in the management of asthma and COPD in SSA. References of the selected original research articles and published review articles were further searched for additional original research articles. We also searched the first 1000 Google scholar searches for original research articles.

Studies included were original research articles that contained information about availability and affordability of any known important medicine and diagnostic test for the management of asthma and COPD, was conducted in SSA between 2000 and March 2018 and published in English language.

Availability of any medicine (s) or diagnostic test (s) was expressed as a percentage of health facilities where it was present at the time of the study. Affordability was expressed as the total number of days’ wages it would cost the lowest paid unskilled government worker to pay for a diagnostic test or a months’ cost of the medicine. This information was documented precisely as reported in the eligible original research article.

The classes of medicines of interest were those belonging to these categories: inhaled SABA, SAMA, LAMA, ICS, SABA-SAMA combinations, LABA–LAMA combinations, ICS–LABA combinations, oral methylxanthines and LTRA. Studies that also contained information about the availability and affordability of spacer devices were also included due to their role in drug delivery especially in young children and elderly patients. The diagnostic tests of interest were spirometry and peak expiratory flow devices.

The following search terms were used: access OR affordability OR pricing OR cost OR availability AND “essential medicines” OR drugs OR therapies OR medicines AND tests OR diagnostic OR imaging AND asthma OR “chronic obstructive pulmonary disease” OR COPD OR “chronic respiratory disorders” AND Africa. The titles and abstracts of all studies were initially assessed for eligibility. Full texts of those studies that initially met the inclusion criteria were obtained and screened by 2 independent reviewers (DK and RES) and then exported to Endnote citation manager. Three extra authors (RN, WW and BK) independently reviewed the selected original research articles for the eligibility and key information about study setting and design and information about availability and affordability using a data extraction form. We excluded original research articles published in other languages other than English, studies whose full texts could not be accessed for full analysis and review articles.

The methodological quality of the identified studies was assessed by 2 independent reviewers (DK and RES) using the Newcastle–Ottawa scale. A maximum score of 6 and 9 was used for the selected cross sectional and case–control and cohort studies respectively [13].

The PRISMA guidelines for the reporting of systematic reviews were followed (Table 1) [14]. This systematic review was registered in PROSPERO (registration number: CRD42018093391).

**Table 1:** Prisma checklist for the systematic review

Section/topic	#	Checklist item	Reported on page #
*Title*			
Title	1	Identify the report as a systematic review, meta-analysis, or both	1
*Abstract*			
Structured summary	2	Provide a structured summary including, as applicable: background; objectives; data sources; study eligibility criteria, participants, and interventions; study appraisal and synthesis methods; results; limitations; conclusions and implications of key findings; systematic review registration number	2–3
*Introduction*			
Rationale	3	Describe the rationale for the review in the context of what is already known	4
Objectives	4	Provide an explicit statement of questions being addressed with reference to participants, interventions, comparisons, outcomes, and study design (PICOS)	5
*Methods*			
Protocol and registration	5	Indicate if a review protocol exists, if and where it can be accessed (e.g., Web address), and, if available, provide registration information including registration number	7
Eligibility criteria	6	Specify study characteristics (e.g., PICOS, length of follow-up) and report characteristics (e.g., years considered, language, publication status) used as criteria for eligibility, giving rationale	5–6
Information sources	7	Describe all information sources (e.g., databases with dates of coverage, contact with study authors to identify additional studies) in the search and date last searched	5
Search	8	Present full electronic search strategy for at least one database, including any limits used, such that it could be repeated	6
Study selection	9	State the process for selecting studies (i.e., screening, eligibility, included in systematic review, and, if applicable, included in the meta-analysis)	6
Data collection process	10	Describe method of data extraction from reports (e.g., piloted forms, independently, in duplicate) and any processes for obtaining and confirming data from investigators	5–6
Data items	11	List and define all variables for which data were sought (e.g., PICOS, funding sources) and any assumptions and simplifications made	5
Risk of bias in individual studies	12	Describe methods used for assessing risk of bias of individual studies (including specification of whether this was done at the study or outcome level), and how this information is to be used in any data synthesis	Not applicable
Summary measures	13	State the principal summary measures (e.g., risk ratio, difference in means)	Not applicable
Synthesis of results	14	Describe the methods of handling data and combining results of studies, if done, including measures of consistency (e.g., I^2^) for each meta-analysis	Not applicable
Risk of bias across studies	15	Specify any assessment of risk of bias that may affect the cumulative evidence (e.g., publication bias, selective reporting within studies)	Not applicable
Additional analyses	16	Describe methods of additional analyses (e.g., sensitivity or subgroup analyses, meta-regression), if done, indicating which were pre-specified	Not applicable
*Results*			
Study selection	17	Give numbers of studies screened, assessed for eligibility, and included in the review, with reasons for exclusions at each stage, ideally with a flow diagram	7
Study characteristics	18	For each study, present characteristics for which data were extracted (e.g., study size, PICOS, follow-up period) and provide the citations	7–11
Risk of bias within studies	19	Present data on risk of bias of each study and, if available, any outcome level assessment (see item 12)	Not applicable
Results of individual studies	20	For all outcomes considered (benefits or harms), present, for each study: (a) simple summary data for each intervention group (b) effect estimates and confidence intervals, ideally with a forest plot	Not applicable
Synthesis of results	21	Present results of each meta-analysis done, including confidence intervals and measures of consistency	Not applicable
Risk of bias across studies	22	Present results of any assessment of risk of bias across studies (see item 15)	Not applicable
Additional analysis	23	Give results of additional analyses, if done (e.g., sensitivity or subgroup analyses, meta-regression [see item 16])	Not applicable
*Discussion*			
Summary of evidence	24	Summarize the main findings including the strength of evidence for each main outcome; consider their relevance to key groups (e.g., healthcare providers, users, and policy makers)	11–13
Limitations	25	Discuss limitations at study and outcome level (e.g., risk of bias), and at review-level (e.g., incomplete retrieval of identified research, reporting bias)	13
Conclusions	26	Provide a general interpretation of the results in the context of other evidence, and implications for future research	14
*Funding*			
Funding	27	Describe sources of funding for the systematic review and other support (e.g., supply of data); role of funders for the systematic review	15

*From:* Moher D, Liberati A, Tetzlaff J, Altman DG, The PRISMA Group (2009). Preferred Reporting Items for Systematic Reviews and Meta-Analyses: The PRISMA Statement. PLoS Med 6(7): e1000097

## Results

**Fig. 1:**
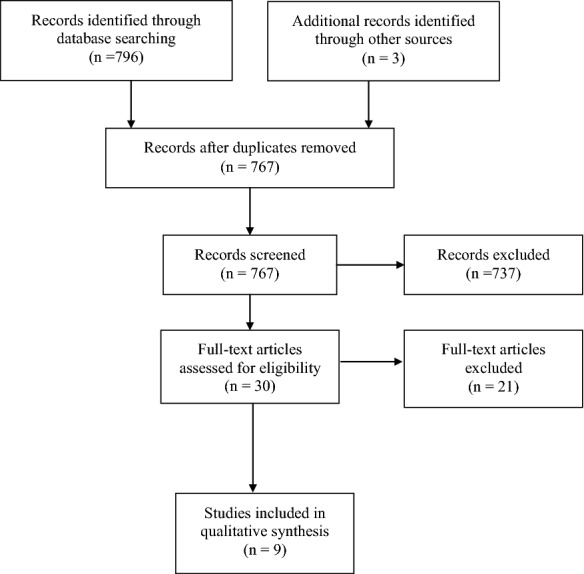
Flow diagram for the systematic review

A total of 796 published articles were identified after searching the 3 databases. Thirty-two duplicates were removed, leaving 764 articles. The titles and abstracts of 764 articles were screened. Of these, 737 articles were excluded because they lacked the information of interest. Full texts of 27 articles were assessed for eligibility and only 6 articles met the inclusion criteria. Three papers were added after searching Google Scholar, references of the eligible original articles and published review articles, making a total of 9 eligible original research articles which were included in the systematic review [15–23] (Fig. 1—Flow diagram summarizing the identification of eligible articles).

### Study characteristics and methodological quality

All eligible studies were cross sectional in nature. Six of the studies (67%) were performed in a single African country (Malawi, Uganda-2 different studies, Nigeria, Ghana, South Africa) [15–17, 19, 20, 23]. The rest were multi-country studies performed in both an African country and other LMIC outside Africa [18, 21, 22]. In these multi-country studies, only data reported from the African countries was obtained.

Only 1 study was assigned the maximum score of 6 [16], with the rest scoring 5 (n = 2, 22.2%) [19, 20] and 4 (n = 6, 66.7%) [15, 17, 18, 21–23] on methodological quality.

The eligible original studies included in the systematic review are summarised in Table 2.

**Table 2:** Summary of the eligible studies included in the systematic review

Study, year and reference	Country (ies) where study was done	No. of health facilities surveyed	No. of essential medicines and diagnostic tests studied	Key study findings about availability and affordability	Method quality score
1. Mendis et al. [15]	6 LMIC (Only one African country included-Malawi).	20 public and 16 private facilities.	2 essential medicines (Salbutamol and Beclometasone inhalers)	Availability of beclometasone: 0% in public sector and 38% in private sector Affordability of salbutamol and beclometasone combination: 9.2 days’ wages	4/6
2. Kibirige et al. [16]	Uganda	23 public and 22 private facilities and 85 private pharmacies	17 essential medicines and 2 diagnostic tests (Spirometry and peak flow-metry)	Availability of inhaled SABA, oral LTRA, ICS–LABA combinations, ICS, oral theophylline, inhaled SAMA, inhaled SAMA and SABA combination and inhaled LAMA monotherapy or with LABA: 75, 60.8, 46.9, 45.4, 16.9, 12.3, 10.8 and 0% respectively Availability of spirometry and peak flow-metry: 24.4% and 6.7% respectively Affordability: inhaled salbutamol-2.2 days’ wages, inhaled beclometasone-5.3 days’ wages, inhaled formeterol-beclometasone-6.4 days’ wages, oral montelukast-6.9 days’ wages, inhaled salmeterol-fluticasone propionate-10.2 days’ wages, inhaled salbutamol-ipratropium-10.7 days’ wages and 17.1 days’ wages for formoterol/budesonide Affordability of spirometry: 27.8 days’ wages	6/6
3. Desalu et al. [17]	Nigeria	68 tertiary public hospitals	6 classes of essential medicines and 2 diagnostic tests (Spirometry and peak flow-metry)	Availability of inhaled anti-cholinergics, oral LTRA, ICS, SABA nebules, ICS–LABA combinations, inhaled SABA and oral theophylline was 2.9%, 5.9%, 23.5%, 35.3%, 50%, 76.5%, 76.5% respectively Availability of spirometry and peak flow-metry was 29.4% and 38% respectively	4/6
4. Babar et al. [18]	52 LMICs (21 SSA countries)	2 private retail pharmacies, 1 national procurement centre and 1 public hospital for each participating country	3 essential medicines (Salbutamol, Beclometasone and Budesonide)	Availability of beclometasone and budesonide: 0% in the surveyed sites in Burundi, Cameroon, Democratic Republic of Congo (DRC), Djibouti, Nigeria, Tanzania and Togo Affordability of innovator budesonide in Burkina Faso, Mozambique and Republic of Guinea was 48 days’ wages, 51 days’ wages and 107 days’ wages respectively Affordability of the lowest priced generic beclometasone was < 2 days’ wages in Kenya, South Africa, Uganda and Zambia and > 2 days’ wages in Ethiopia, Madagascar, Malawi, Sudan and Zimbabwe Affordability of the lowest priced generic salbutamol was < 2 days’ wages in Burkina Faso, DRC, Kenya, South Africa, Tanzania, Uganda, Zambia and Zimbabwe and ≥ 2 days’ wages in Benin, Burundi, Cameroon, Ethiopia, Republic of Guinea, Madagascar, Malawi, Mali, Mozambique and Togo	4/6
5. Nyarko et al. [19]	Ghana	23 health facilities (92%-public and 8%-private)	3 essential medicines (Salbutamol inhaler, Ipratropium bromide and beclometasone inhaler) and 1 diagnostic test (peak flow-metry)	Availability of ipratropium bromide, beclometasone inhaler and salbutamol inhaler was 4.5, 17.4 and 39.1% respectively Availability of peak flow-metry was 13%	5/6
6. Armstrong-Hough et al. [20]	Uganda	196 health facilities	2 essential medicines (Beclometasone and salbutamol inhalers)	Availability of beclometasone and salbutamol inhalers was 1.5% and 19.9% respectively	5/6
7. Cameron et al. [21]	36 LMICs (11 SSA countries)	1 main public hospital, 4 randomly-selected public medicine outlets and 1 private facility for each participating country	1 essential medicine (Salbutamol inhaler)	Mean availability of lowest priced generic salbutamol in 8 SSA countries was 14% (0–55.9%) and 47% (0–95%) in the public and private sector respectively Affordability of lowest priced generic salbutamol in the public sector was a mean of 1.6 days’ wages. In the private sector, the lowest priced generic and innovator salbutamol cost a mean of 2.5 and 4.4 days’ wages respectively	4/6
8. Mendis et al. [22]	8 LMICs (3 SSA countries-Benin, Eriteria and Sudan)	30 health facilities.	3 essential medicines (beclometasone, salbutamol and ipratropium bromide inhalers)	Availability of beclometasone inhaler in Benin, Sudan and Eriteria was 16.7, 21.4 and 33.3% respectively Availability of salbutamol inhaler in Benin, Sudan and Eriteria was 33.3, 71.4 and 100% respectively Availability of ipratropium bromide was 0% in Benin and Eriteria and 14.3% in Sudan	4/6
9. Mash et al. [23]	South Africa	46 primary care facilities	1 diagnostic test (peak flow-metry) Details of essential medicines studied were not given	Availability of peak flow-metry was 53.6%	4/6

*LMIC* Low-and middle-income countries, *SSA* sub-Saharan Africa, *SABA* short acting beta agonists, *SAMA* short acting anti muscarinic agents, *LAMA* long acting anti muscarinic agents, *LABA* long acting beta agonists, *ICS* inhaled corticosteroid, *LTRA* leukotriene receptor antagonists

### Availability of essential medicines used in management of exacerbations of both asthma and COPD (inhaled SABA and SAMA monotherapy)

#### Inhaled SABA monotherapies

Six studies evaluated the availability of inhaled SABA (inhaled salbutamol) [16, 17, 19–22] which ranged from 19.9% in one study performed in Uganda [20] to 100% in Eriteria [22]. Low rates of availability of inhaled SABA were reported in a multi-centre study of 8 SSA countries (14% and 47% in public and private sector respectively) [21], Benin (33.3%) [22] and Ghana (39.1%) [19]. Comparable rates of moderate availability were reported in another study in Uganda (75%) [16] and other studies performed in Sudan (71.4%) [22] and Nigeria (76.5%) [17].

#### Inhaled SAMA monotherapy

Availability of inhaled SAMA (ipratropium bromide) was documented to be very low, with levels of 0% in Benin and Eriteria [22], 2.9% in Nigeria [17], 4.5% in Ghana [19], 12.3% in Uganda [16] and 14.3% in Sudan [22].

### Availability of medicines used in symptom control of both asthma and COPD (ICS, ICS–LABA combinations, low dose theophylline, LTRA and inhaled tiotropium (LAMA)

#### Ics

Availability of ICS reported by 7 studies [15–20, 22] ranged from 0% in a multicenter study of 7 SSA countries [18] and in another study performed in Malawi [15] to 45.5% in Uganda [16]. Very low availability of ICS was noted in another study performed in Uganda (1.5%) [20], a multi-country study involving 3 SSA countries (Benin-16.7%, Sudan-21.4% and Eriteria-33.3%) [22], Ghana (17.4%) [19], 23.5% (Nigeria) [17] and the private sector in Malawi (38%) [15].

#### Inhaled ICS–LABA combinations

Only 2 studies performed in Uganda [16] and Nigeria [17] assessed availability of ICS–LABA combinations; documenting comparable low rates of 46.9% and 50% respectively.

#### Oral low dose theophylline and LTRA

Varying levels of availability of oral theophylline and LTRA were reported by 2 studies [16, 17]. In a study performed in Uganda, availability of oral theophylline and LTRA was 16.9% and 60.8% respectively [16] compared to 76.5% and 5.9% respectively in a study performed in Nigeria [17].

#### Inhaled LAMA (tiotropium)

Only 1 study investigated availability of inhaled tiotropium, reporting its absence in all health facilities surveyed [16].

### Availability of medicines used in symptom control of COPD only (inhaled LABA monotherapy, SABA–SAMA combinations and LAMA and LABA combinations)

Availability of all the above medicines was studied by only 1 study, documenting comparable very low levels of availability of inhaled LABA monotherapies and SABA–SAMA combinations of 10% and 10.8% respectively. No surveyed health facility had any inhaled LABA–LAMA combination [16].

No study investigated the availability of LABA–LAMA–ICS triple combinations, pneumococcal vaccines and phosphodiesterase 4 inhibitors which are essential medicines in the management of COPD.

### Spacers

Due to the important role of spacers in drug delivery in the management of asthma and COPD in children and elderly patients, we also included information about their availability from 4 studies [16, 17, 19, 23]. With the exception of a study performed in South Africa where spacers were available in 72.9% of the surveyed 46 primary healthcare facilities, the rest of the studies reported very low levels of availability of 0% in Ghana [19], 18.5% and 19.2% for adult and paediatric spacers respectively in Uganda [16] and 20.6% in Nigeria [17].

### Availability of diagnostic tests for asthma and COPD

Only 4 studies contained findings about the availability of the diagnostic tests [16, 17, 19, 23]. Availability of peak expiratory flow devices was reported about in all the 4 studies (6.7% in Uganda, 13% in Ghana, 38% in Nigeria and 53.6% in South Africa). Availability of spirometry was reported only by studies performed in Uganda (24.4%) [16] and Nigeria (29.4%) [17].

### Affordability of medicines used in management of exacerbations of both asthma and COPD (inhaled SABA and SAMA monotherapies)

#### Inhaled SABA (salbutamol)

In a multi-centre study by Babar et al. [18] that included 22 countries in SSA, the lowest priced generic (LPG) inhaled salbutamol cost < 2 days’ wages in 8 countries (Burkina Faso, Democratic Republic of Congo, Kenya, South Africa, Tanzania, Uganda, Zambia and Zimbabwe) and ≥ 2 days’ wages in 10 countries (Benin, Burundi, Cameroon, Ethiopia, Republic of Guinea, Madagascar, Malawi, Mali, Mozambique and Togo). Another multi-centre study that included information from 8 countries in SSA reported the lowest priced generic (LPG) salbutamol inhaler to cost a mean of 1.6 days’ wages and 2.5 days’ wages in the public and private sector respectively. The innovator brand of salbutamol inhaler cost a mean of 4.4 days’ wages [21]. In another study done in Uganda, the LPG salbutamol inhaler cost 2.2 days’ wages [16].

#### Inhaled SAMA monotherapy (ipratropium)

The LPG SAMA (inhaled ipratropium) as reported by only 1 study cost 13.7 and 10.7 days’ wages for the 20 µg and 40 µg respectively [16].

### Affordability of medicines used in symptom control of both asthma and COPD (ICS, ICS–LABA combinations, low dose theophylline, LTRA and inhaled LAMA)

#### Ics

A disparity in the affordability of generic and innovator ICS was observed in the study by Babar et al. [18] that included information from 21 countries in SSA. The LPG beclometasone cost < 2 days’ wages in Kenya, South Africa, Uganda and Zambia and ≥ 2 days’ wages in Ethiopia, Madagascar, Malawi, Sudan and Zimbabwe. The cost of innovator budesonide was 48 days’ wages, 51 days’ wages and 107 days’ wages in Burkina Faso, Mozambique and Republic of Guinea respectively. In Uganda, the LPG inhaled beclometasone, fluticasone propionate and budesonide cost 5.3 days’ wages and 8 days’ wages respectively [16].

#### ICS–LABA combinations, SAMA–SABA combinations, low dose theophylline and LTRA

Only 1 study contained information about the affordability of the above medicines [16]. Regarding ICS–LABA combinations, the reported cost of the LPG formoterol/beclometasone, salmeterol/fluticasone propionate and formoterol–budesonide was 6.4 days’ wages, 10.2 days’ wages and 17.1 days’ wages respectively. Inhaled SAMA–SABA (ipratropium–salbutamol combination) cost 10.7 days’ wages while oral LTRA and low dose theophylline both cost 6.9 days’ wages. Adult and paediatric spacers cost 12.9 and 7.5 days’ wages respectively [16].

### Affordability of diagnostic tests of asthma and COPD

One study evaluated affordability of spirometry which cost up to 27.8 days’ wages [16].

## Discussion

To our knowledge, this systematic review provides the first comprehensive assessment of availability and affordability of internationally recommended medicines and diagnostic tests in management of asthma and COPD in SSA. It also evidently demonstrates that studies investigating this key area in SSA are still limited and availability of affordable medicines and diagnostic tests still remains a substantial challenge in clinical practice in the region.

We noted considerable heterogeneity in the study results and health facilities surveyed. Poor availability was widely noted with SAMA, SAMA–SABA combinations, ICS, LAMA, LAMA–LABA combinations and diagnostic tests (spirometry and peak expiratory flow devices). The paucity of studies investigating access to LAMA and LAMA–LABA combinations, which are vital medicines in reducing COPD symptoms and future exacerbations is also revealing. This poor access to medicines for asthma and COPD has been highlighted in SSA and other LMIC and it carries substantial public health implications [24–27]. Availability of SABA (salbutamol inhaler) and LABA–ICS combination was > 70% in Uganda, Nigeria, Sudan and Eriteria [16, 17, 22] and > 45% in Uganda and Nigeria [16, 17]. The fairly good availability could explain the frequent use of SABA that has been described in patients with asthma and COPD in clinical practice.

Poor access to spirometry as a cornerstone for diagnosis and assessment of treatment response in patients with asthma and COPD as noted by this systematic review still remains a challenge. The poor availability of spirometry in health facilities and inadequate proficiency in interpretation of spirometric readings by healthcare workers in clinical practice has been widely documented as an impediment to optimal management of asthma and COPD in SSA [26–28]. The absence of adequate numbers of skilled personnel to perform spirometry and interpret its findings could also partly explain its low availability in SSA.

A dearth of studies about affordability of the medicines and diagnostic tests for asthma and COPD in SSA is also of great concern. The majority of the medicines (controller therapies) especially in the private sector and innovator brands remain unaffordable for most patients in SSA.

Several reasons could explain the poor availability and high costs of the majority of the medicines and diagnostic tests in SSA. There exists a knowledge-practice gap among healthcare practitioners in SSA resulting into patterns of clinical practice that are not in conformity with international treatment guidelines [27, 29]. Low prescribing rates of these medicines by healthcare practitioners directly or indirectly influence their availability both in the public and private sector. Exclusion of these medicines from the national essential medicine lists (EML) and treatment guidelines could also explain the poor availability in SSA. One survey that investigated the number of asthma and COPD essential medicines on the national EML of 32 LMIC (including some countries in SSA) noted that the median number of essential medicines was 7, with a range of 0–22. Notably, no African country included LABA to their national EML. Few low income countries (LIC) included inhaled anti cholinergic agents (20%). None of the LIC included therapies recommended in step 4 and 5 of asthma management by the GINA guidelines. Only a third of them included at least 1 medicine recommended in the step 2 and 3 of COPD management by the GOLD guidelines [30].

The high cost of the medicines and diagnostic tests in SSA could be explained by lack of local price regulatory frameworks or legislation, absence or poor access to national health insurance schemes that can provide the medicines at subsided fees, low scale of local pharmaceutical production of generic medicines and absence of public and private co-financing initiatives to reduce costs of drugs.

### Strengths and limitations of the systematic review

One significant strength of this systematic review is being the first review to offer comprehensive information about the extent of availability and affordability of medicines and diagnostic tests of asthma and COPD in SSA. Despite this, some of the limitations are heterogeneity of the study findings and health facilities surveyed and the low methodological quality of the eligible original studies.

## Conclusion

A better understanding of the magnitude and reasons to explain the challenge of poor availability and high cost of these medicines and diagnostic tests in SSA is important to guide better implementation of pragmatic solutions and guidelines. Poor availability and unaffordability of medicines and diagnostic tests recommended for the management of asthma and COPD in SSA could be addressed through increasing awareness about the burden of both conditions and their optimal management among healthcare practitioners, improving local manufacturing of cheap good quality generic medicines, updating national EML and treatment guidelines, improving supply chain and forecast and sustained and equitable government financing of health budgets. Implementation of health policies like national health insurance schemes, regulation of local retail prices of chronic diseases and introduction of preferential registration procedures for locally manufactured generic drugs by drug regulatory institutions can help address the challenge of high costs of medicines of asthma and COPD in SSA.

Due to differences in economic status of countries in SSA, more robust country-specific large studies about access to affordable essential medicines and diagnostic tests are needed to further appreciate the magnitude of this public health problem in this region.

## Abbreviations

CRD: chronic respiratory disorders
COPD: chronic obstructive pulmonary disease
LMIC: low-and middle income countries
GBD: Global Burden of Diseases
SSA: sub-Saharan Africa
GINA: Global Initiative for Asthma
GOLD: Global Initiative for Chronic Obstructive Lung Disease
SABA: short acting beta agonists
SAMA: short acting anti muscarinic agents
LAMA: long acting anti muscarinic agents
LABA: long acting beta agonists
ICS: inhaled corticosteroid
LTRA: oral leukotriene receptor antagonists
